# Clinical implications of quantitative real-time RT–PCR analysis of *hTERT* gene expression in human gliomas

**DOI:** 10.1038/sj.bjc.6600754

**Published:** 2003-02-18

**Authors:** A Tchirkov, C Rolhion, J-L Kémény, B Irthum, S Puget, T Khalil, O Chinot, F Kwiatkowski, B Périssel, P Vago, P Verrelle

**Affiliations:** 1 Département de Radiothérapie, Centre Jean Perrin, 63011 Clermont-Ferrand, France; 2 Service de Cytogénétique Médicale, Hôpital Gabriel Montpied, 63001 Clermont-Ferrand, France; 3 INSERM U484, 63005 Clermont-Ferrand, France; 4 Laboratoire d'Anatomie Pathologique, Hôpital Gabriel Montpied, 63001 Clermont-Ferrand, France; 5 Services de Neurochirurgie, Hôpital Gabriel Montpied, 63001 Clermont-Ferrand, France; 6 Service de Neurochirurgie, Hôpital de la Timone, 13385 Marseille, France; 7 Service Statistiques et Communications Médicales, Centre Jean Perrin, 63011 Clermont-Ferrand, France

**Keywords:** *hTERT*, real-time PCR, glioblastoma multiforme, diagnosis, prognosis

## Abstract

The presence of telomerase activity in a glioma may be a predictor of its malignant potential. Activation of telomerase is regulated at the transcriptional level of the human telomerase reverse transcriptase (*hTERT*). Here, we evaluated whether the amount of *hTERT* mRNA provides a molecular marker of glioma malignancy that would have clinical utility. We used a real-time RT–PCR to assess the number of *hTERT* transcripts in primary tumour samples derived from 70 glioma patients. Results were standardised by quantifying the number of *ABL* transcripts as internal control and expressed as *hTERT/ABL* ratio. The percentage of patients with detectable *hTERT* mRNA markedly increased with enhanced malignancy: low-grade gliomas expressed *hTERT* in one out of 14 cases (7.1%), anaplastic gliomas in four out of 13 cases (30.8%) and glioblastoma multiforme (GBM) tumours in 30 out of 43 cases (69.8%). The mean *hTERT/ABL* ratio was significantly higher in GBMs than in non-GBMs. Subdividing *hTERT/ABL* ratios as low (⩽25%) and high (>25%), we found that the overall survival among *hTERT*-positive GBMs was significantly worse in high *hTERT* expressors than in low *hTERT* expressors (*P*=0.0082). We conclude that the amount of *hTERT* mRNA may represent a diagnostic and prognostic indicator for GBM patients.

Gliomas are the most common of primary brain tumours in humans and form a complex group because of the great variability in degree of malignancy that exists among them. The grade of malignancy is one of the major factors in the prognosis of glioma patients (DeAngelis, 2001). Patients with low-grade tumours typically survive more than 5 years, patients with anaplastic tumours about 3 years, and patients with glioblastoma multiforme (GBM), glioma of the highest grade of malignancy, have the poorest prognosis with median survival of less than 1 year despite modern therapy. Molecular genetic research has led to an increased knowledge of markers that are associated with glioma malignancy and may be useful diagnostic and therapeutic targets. In particular, malignant progression in gliomas has been correlated with the presence of telomerase activity in tumour cells (Langford *et al*, 1995).

Telomerase is a specialised reverse transcriptase enzyme that maintains telomeres by adding telomeric TTAGGG repeats to the ends of human chromosomes (Morin, 1989). Telomerase is repressed in most normal human somatic cells, and without new synthesis of telomeres the chromosomes shorten with progressive cell division, eventually triggering either replicative senescence or apoptosis when telomere length becomes critically short (Harley *et al*, 1990). In contrast, cells that express a sufficient level of telomerase activity escape from replication limitations and are considered as immortal. Reactivation of telomerase is believed to be an essential step during malignant tumour progression (Counter *et al*, 1994). A strong association between the presence of telomerase and malignancy has been established in nearly all cancer types (reviewed in Shay *et al*, 2001). Importantly, high telomerase activity is generally associated with high tumour aggressiveness.

Studies of gliomas have shown that more than 50% of tumours have telomerase activity, detection rates increasing with the grade of malignancy (Langford *et al*, 1995; Nakatani *et al*, 1997; Le *et al*, 1998; Sugita *et al*, 2000). These observations have suggested that the presence of such activity may indicate the malignant potential of a glioma. However, the prognostic value of telomerase in gliomas remains uncertain. Although telomerase activity has been correlated with inferior survival in low-grade and anaplastic astrocytomas, no clear relation has been determined between the detection or relative level of telomerase activity and patient survival in GBMs, the most common and most malignant gliomas (Nakatani *et al*, 1997; Hiraga *et al*, 1998; Huang *et al*, 1999; Kleinschmidt-Demasters *et al*, 2000).

The activation of telomerase is tightly regulated at the transcriptional level of the human telomerase reverse transcriptase (*hTERT*). Many studies have suggested that the transcription of *hTERT* represents the rate-limiting step in telomerase expression, and the detection of *hTERT* transcripts using RT–PCR revealed a strong correlation with telomerase activity in the majority of tumours (reviewed in Pool *et al*, 2001). A recent method of real-time PCR has greatly facilitated sensitive and quantitative detection of *hTERT* mRNA. In this study, we evaluated the level of *hTERT* transcripts in 70 primary gliomas and correlated them with malignancy grade. In GBMs, we also studied the relation between the amount of *hTERT* mRNA and patient survival. We found that *hTERT* expression increased with malignancy grade and was highly associated with GBMs. Within this important glioma subgroup, the level of *hTERT* mRNA was predictive of patient survival.

## MATERIALS AND METHODS

### Patients and samples

Primary tumour samples were obtained from 70 glioma patients at surgical resection of their lesion. The samples for *hTERT* expression analysis were taken from the material of resection during the course of standard diagnostic procedure. According to the French law on biomedical research, this is an epidemiological study that does not have to be submitted to an Institutional Review Board. Immediately after surgery, the samples were snap-frozen and stored in liquid nitrogen until RNA extraction.

Histological diagnosis and grading of tumours were consistent with the WHO criteria (World Health Organization, 2000). A total of 14 tumours were classified as low-grade gliomas, 13 tumours were anaplastic gliomas, and the remaining 43 tumours were classified as GBMs. The details of tumour type and grade are given in Table 1

**Table 1:** Detection of *hTERT* mRNA in gliomas of varying type and malignancy grade

			***hTERT* mRNA**	
**Type of glioma**	**WHO grade**	**Number of cases**	**Positive**	**Negative**
*Low-grade gliomas*		14	1 (7.1%)	13 (92.9%)
Pilocytic astrocytoma	I	3	0	3
Diffuse astrocytoma	II	2	0	2
Oligoastrocytoma	II	3	0	3
Oligodendroglioma	II	6	1	5
				
*Anaplastic gliomas*		13	4 (30.8%)	9 (69.2%)
Anaplastic astrocytoma	III	2	0	2
Anaplastic oligoastrocytoma	III	3	0	3
Anaplastic oligodendroglioma	III	8	4	4
				
*Glioblastoma multiforme*	IV	43	30 (69.8%)	13 (30.2%)

.

Among 70 glioma patients, 46 patients were male and 24 were female. The mean age at diagnosis was 31.5±12.2 years for patients with low-grade tumours, 43.7±12.7 years for patients with anaplastic gliomas and 59.5±10.7 years for patients with GBMs. The median follow-up was 61 months (range, 32–184 months). In two GBM cases, very early postsurgery death occurred. In most cases, postsurgical treatments included radiotherapy and/or various chemotherapy regimens. Note that in our GBM series, postsurgical treatments had no prognostic impact (data not shown).

### Extraction of RNA and preparation of cDNA

Total RNAs were extracted from tumour samples using Trizol® reagent (GIBCO/BRL, Grand Island, NY, USA), according to the manufacturer's instructions. Sample RNA (1 *μ*g) was reverse transcribed as reported elsewhere (Rolhion *et al*, 1999). In parallel, reverse transcription reaction was set up without enzyme and then the sample was processed to PCR as a control for DNA contamination.

### Quantitative real-time RT–PCR for *hTERT* mRNA

**Figure 1:**
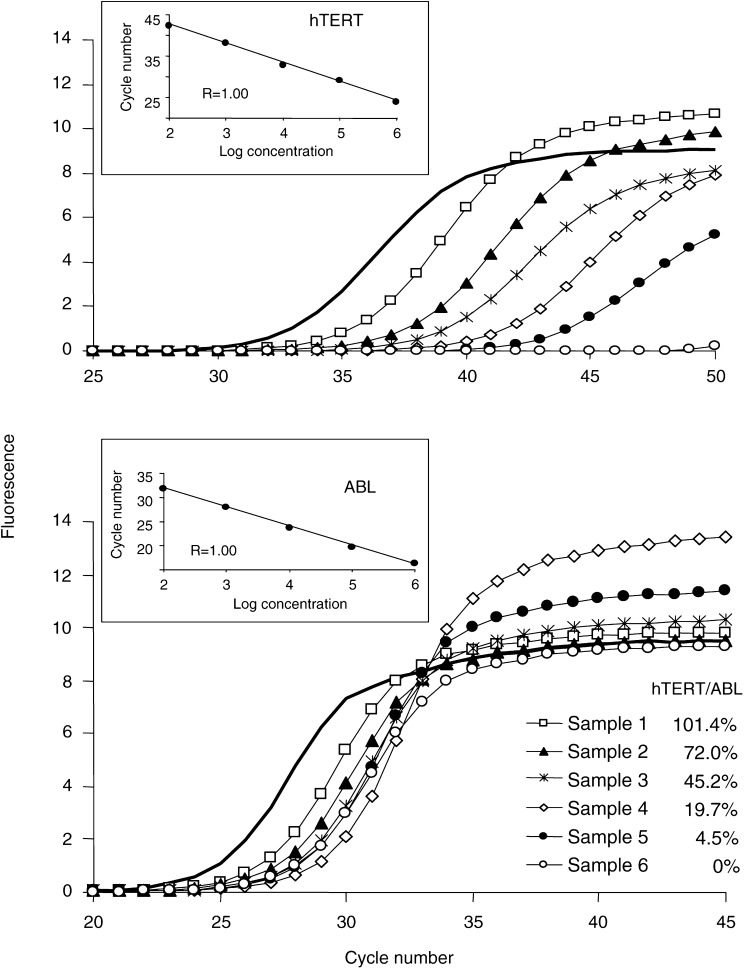
Example of real-time PCR quantification of *hTERT* in tumour samples. Amplification profiles obtained on the LightCycler are presented for *hTERT* (upper graph) and *ABL* (lower graph) transcripts. Linear regressions of standard dilution series, indicating accuracy of the analysis, are shown on the upper left part of each graph. Calculation of the number of *hTERT* and *ABL* transcripts was done by using these standard curves. One of the standards was included in each PCR run (amplification profiles shown as solid lines, 10^5^ molecules). For each sample, the number of *hTERT* transcripts was divided by the number of *ABL* transcripts in order to standardise the level of *hTERT* mRNA. The obtained ratios expressed as percentage are shown.

We assessed the number of *hTERT* mRNA transcripts using real-time PCR in the LightCycler system (Roche Diagnostics, Meylan, France). The amplification of *hTERT* mRNA was performed with the DNA Master SYBRGreen I reagent set (Roche Diagnostics, Meylan, France) using the following primers: forward primer 5′-GGAGCAAGTTGCAAAGCATTG-3′ and reverse primer 5′-TCCCACGACGTAGTCCATGTT-3′. Quantification of *ABL* transcripts as an internal control for the amount and quality of cDNA was performed for all samples, using the forward primer 5′-GCCGCTCGTTGGAACTCCAAGG-3′ and reverse primer 5′-TGACTGGCGTGATGTAGTTTGCTT-3′ (Tchirkov *et al*, 1998). Several studies have confirmed that the *ABL* gene is an appropriate reference in quantitative real-time PCR studies of both pathological and normal cells (Buonamici *et al*, 2002; Moniotte *et al*, 2001). During initial optimisation of PCR conditions, amplified products were analysed using agarose gels to ensure correct product size. Once the PCR product size was verified, the melting temperature of the product determined with LightCycler melting-curve analysis was used to control for the specificity of amplifications. The number of transcripts in samples was calculated with the LightCycler software, using the calibration data obtained with serial dilutions of purified PCR products containing known numbers of cDNA molecules of each gene (Figure 1

). The results of real-time PCR were given as the ratio between *hTERT* and *ABL* transcripts, expressed as a percentage. Glioma samples were analysed in a blind-trial fashion. All experiments were performed in triplicate, with good consistency of results (the mean coefficient of variation was 8.4%).

### Statistical analysis

The overall survival time was calculated in months after initial surgery date. For patients with *hTERT-*expressing GBMs, a receiver operating characteristic (ROC) analysis was used to determine which *hTERT* level best discriminated between those patients who did not reach the median survival time (15 months for *hTERT*-positive GBM patients) and the others (Bamber, 1975; Hanley and McNeil, 1982). This method was valid since all patients had died by the time of analysis. Kaplan–Meier estimation was used to evaluate the overall survival and the log-rank statistics was used to compare survival between subgroups of patients (Kaplan and Meier, 1958). Other analyses of statistical links between biological and clinical parameters were performed using standard tests.

## RESULTS

### hTERT mRNA expression in gliomas

We studied 70 gliomas for the presence of *hTERT* mRNA using a real-time RT–PCR. Table 1 shows that low-grade gliomas expressed *hTERT* in one out of 14 cases (7.1%), anaplastic gliomas in four out of 13 cases (30.8%) and GBMs in 30 out of 43 cases (69.8%). Thus, the frequency of *hTERT* expression significantly increased with advanced glioma malignancy (*χ*^2^-test, *P*=0.00024). Moreover, on comparing GBMs and non-GBMs, we found that the expression of *hTERT* was highly associated with GBMs (χ^2^-test, *P*=0.00003). Among non-GBMs, the expression of *hTERT* was detected mainly in anaplastic oligodendrogliomas.

**Figure 2:**
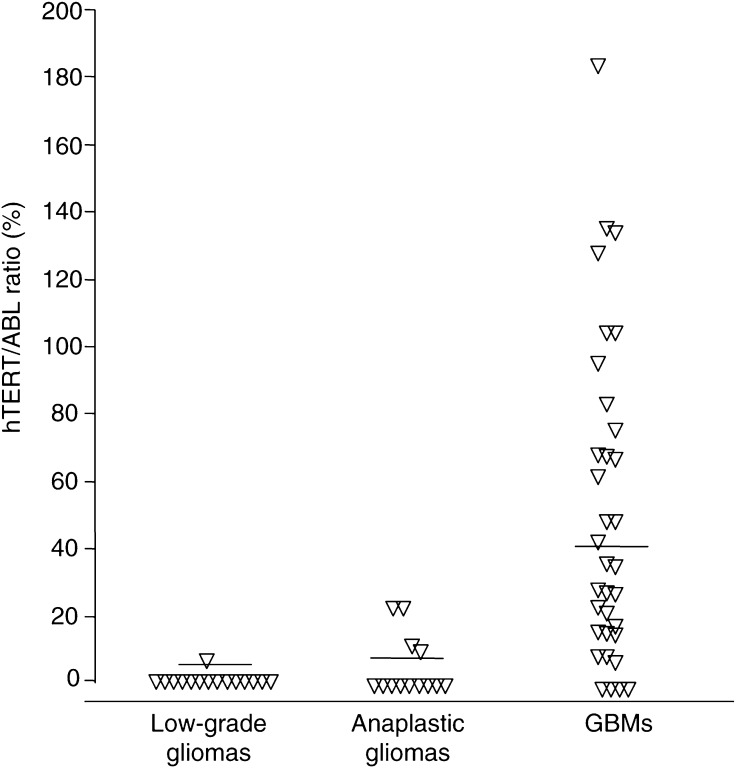
Individual *hTERT*/*ABL* ratios (%) determined in glioma samples according to the investigated tumour types. The mean values are shown as horizontal bars for each group: low-grade tumours (0.2%), anaplastic gliomas (3.7%), GBMs (37.8%).

In *hTERT*-positive glioma samples, the number of *hTERT* transcripts was assessed and normalised to the expression of a housekeeping gene, *ABL*. Among glioma patients with detectable *hTERT* mRNA, the *hTERT/ABL* ratio ranged between 2.6 and 180.1% (Figure 2

). The *hTERT/ABL* ratio was significantly different between the low-grade, anaplastic and GBM tumour groups (Kruskal–Wallis test, *P*=0.00013). Among patients with detectable levels of *hTERT* mRNA, the amount of *hTERT* transcripts was significantly higher among the GBM patients (54.2%, mean) than in non-GBMs (10.2%, mean) (Mann–Whitney *U*-test, *P*=0.012). Overall, the level of *hTERT* expression was neither age- (Spearman rank test, NS) nor gender-related (Kruskal–Wallis test, NS).

### Prognostic significance of hTERT expression in GBMs

**Figure 3:**
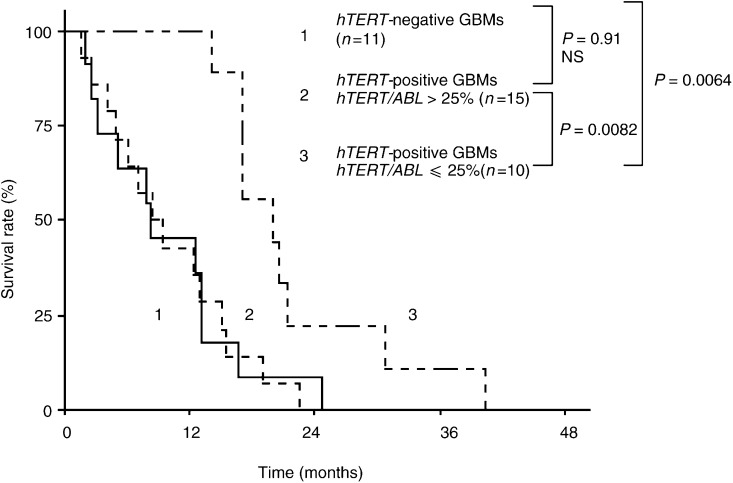
Survival of GBM patients in relation to *hTERT* status in their tumours. Patients, whose tumours expressed *hTERT* at low levels, survived significantly longer (19 months, median) than those patients who showed high *hTERT* levels in their tumours (8 months, median) or than *hTERT*-negative patients (8 months, median) did. Note the very similar survival period for high *hTERT* expressors and *hTERT*-negative patients.

In the GBM group, we tested for a possible relation between the relative level of *hTERT* expression and patient survival. The cases with gross total tumour excision (*n*=5), where no residual contrast enhancement was detected on early postoperative computed tomography scans, were excluded from analysis, as complete resection of the lesion could influence favourably the survival in GBMs (Scott *et al*, 1999). Among the 36 patients with subtotal resection of their lesion, 25 had *hTERT* positive tumours. In the *hTERT*-positive patient group, we determined, using an ROC analysis, a cut-off level of *hTERT* transcripts of 23.7% that best segregated patients into poor- and good-prognosis subgroups (as described in the Statistical Analysis). We found that the patients with high (>25%) *hTERT* mRNA levels had significantly shorter survival *vs* the patients with low (⩽25%) levels (log-rank test, *P*=0.0082, Figure 3

). The 11 patients with non-detectable *hTERT* mRNA had survival equivalent to that of high *hTERT* expressors (log-rank test, *P*=0.91, NS). The survival of these patients was also significantly worse than that of patients with low *hTERT* levels (log-rank test, *P*=0.0064).

## DISCUSSION

In this study, we measured the *hTERT* mRNA level in tumour specimens from a cohort of 70 patients with gliomas using a recently introduced real-time quantitative PCR technique. We found a progressive increase in the *hTERT* detection rate with increasing grade of glioma malignancy: 7% for low-grade gliomas, 31% for anaplastic gliomas and 70% for GBMs. These data are in agreement with the detection rates of telomerase activity reported for these tumour types (Hiraga *et al*, 1998; Le *et al*, 1998; Harada *et al*, 2000; Sugita *et al*, 2000). The association of *hTERT* expression with GBMs was highly specific when comparing GBMs to non-GBMs. This observation is in line with the recent demonstration of a similar association at the level of *hTERT*-protein overexpression (Chakravarti *et al*, 2001). Among non-GBMs, *hTERT* mRNA was detectable in anaplastic oligodendrogliomas, consistent with a previous report (Langford *et al*, 1995). Among patients with detectable levels of *hTERT* mRNA, the amount of *hTERT* transcripts was significantly higher among the GBM patients. Taken together, these findings suggest that the level of *hTERT* mRNA may be used as an indicator of enhanced glioma malignancy. The *hTERT* analysis may complement histology and help to refine tumour grading and classification.

Current system of pathological grading for human gliomas is often nonprognostic: some tumours responding well to treatment may be histologically indistinguishable from nonresponding ones. Previous studies have suggested that telomerase activity in gliomas may have utility in tumour prognosis, as the presence of such activity has been correlated with a poor prognosis for low-grade and anaplastic tumours (Nakatani *et al*, 1997; Hiraga *et al*, 1998; Huang *et al*, 1999). However, studies have failed to identify any significant relation between the level of telomerase activity and patient survival in GBMs (Nakatani *et al*, 1997; Hiraga *et al*, 1998; Kleinschmidt-Demasters *et al*, 2000). In the present study, we found that among *hTERT*-positive GBM patients, the overall survival was significantly worse in the patients with high levels of *hTERT* mRNA. This finding is compatible with the result regarding *hTERT*-protein overexpression in GBMs, which showed that high levels of the protein correlated with reduced patient survival (Chakravarti *et al*, 2001). Importantly, the patients without detectable *hTERT* mRNA had a short survival equivalent to that of high *hTERT* expressors. In contrast, the patients with low levels of *hTERT* had prolonged survival. Thus, the level of *hTERT* mRNA may predict decreased or increased survival in GBMs.

The level of *hTERT* mRNA estimated with real-time RT–PCR procedure is the average amount of transcripts in a whole tumour sample and mainly depends on the number of *hTERT*-positive cells present in the tumour tissue. Studies of *hTERT*-protein distribution using immunohistochemistry in cultured cells and tissue sections have shown that *hTERT* expression was detected in almost all neoplastic cells in cancer tissues with high telomerase activity, whereas cancers with low telomerase activity had fewer *hTERT*-positive cancer cells (Hiyama *et al*, 2001). The burden of *hTERT*-positive cells probably reflects the degree of expansion of clonogenic tumour cell population, which has a great selective advantage and proliferation capacity provided by telomerase activity. It is possible that the level of expansion correlates with the size of clonogenic cell fraction. Thus, low *hTERT* expressors might survive longer than high *hTERT* expressors since they are likely to have fewer neoplastic stem cells at the time of diagnosis, and cytotoxic treatments are in this case more efficient. Nevertheless, testing this hypothesis requires further studies of *hTERT*-positive gliomas combining the evaluation of *hTERT*-protein expression at the individual cell level and clonogenic assays.

Equally poor prognosis found for GBM patients in the groups with high *hTERT* expression and without *hTERT* expression points to the fact that aggressive growth of some GBMs may occur in the absence of telomerase. Telomerase-negative GBMs might achieve immortalisation by an alternative mechanism of telomere length stabilisation. Evidence in support of this hypothesis has been reported in a study of telomere length in gliomas (Morii *et al*, 1997). Compared with telomerase-positive gliomas, telomerase-negative gliomas were found to have very long and heterogeneous telomeres, characteristics seen in tumour cells that have acquired an alternative mechanism for lengthening their telomeres. The nature of this mechanism named alternative lengthening of telomeres (ALT) and described in several tumour types is currently unclear, but may involve nonreciprocal recombination between telomeres (Bryan *et al*, 1997). Given the poor prognosis in *hTERT*-negative patients, it seems important to investigate the possible role of the ALT mechanism in malignant progression of GBMs.

In conclusion, *hTERT* mRNA expression may be used as a molecular marker of glioma malignancy that may be particularly helpful in diagnosing GBMs as a complement to existing approaches. In addition, the level of *hTERT* transcripts appears to predict in GBMs decreased or increased survival. In the development and future application of anti-telomerase treatments of malignant gliomas (Komata *et al*, 2002), *hTERT* analysis of any given tumour will be essential.
